# Dentigerous cyst and the importance of early detection. report of a pediatric case

**DOI:** 10.21142/2523-2754-1002-2022-111

**Published:** 2022-06-27

**Authors:** Olger Jesús Benavides-Guzmán, Alejandro Pineda Méndez, Yalil Augusto Rodríguez-Cárdenas, Arón Aliaga-Del Castillo, Gustavo Armando Ruíz-Mora

**Affiliations:** 1 Division of Oral and Maxillofacial Radiology, School of Dentistry, Universidad Científica del Sur. Lima, Peru. olgerjesus1995@hotmail.com, dentalmax_vincentt@live.com.mx, yalilrodriguez@gmail.com Universidad Científica del Sur Division of Oral and Maxillofacial Radiology, School of Dentistry Universidad Científica del Sur Lima Peru olgerjesus1995@hotmail.com dentalmax_vincentt@live.com.mx yalilrodriguez@gmail.com; 2 Department of Orthodontics, Bauru Dental School. Universidade of Sao Paulo, Brazil. a_aliaga@hotmail.com Universidade Federal de São Paulo Department of Orthodontics, Bauru Dental School Universidade of Sao Paulo Brazil a_aliaga@hotmail.com; 3 Division of Orthodontics, Faculty of Dentistry, Universidad Nacional de Colombia. Bogota, Colombia. garruiz@gmail.com Universidad Nacional de Colombia Division of Orthodontics, Faculty of Dentistry Universidad Nacional de Colombia Bogota Colombia garruiz@gmail.com

**Keywords:** dentigerous cyst, pediatric, cone beam computed tomography, developmental cyst, quiste dentígero, pediátrico, tomografía computarizada de haz cónico, quiste del desarrollo

## Abstract

Dentigerous cysts are a common cystic pathology that develop between the first and third decade of life and are mainly associated with impacted or erupted mandibular third molars followed by maxillary canines and maxillary third molars. These kinds of cysts are the result of the proliferation of enamel epithelium after its formation, the pathogenesis of which is not clear. Few of these cysts have been reported in pediatric patients. The following case report presents the rare occurrence of a dentigerous cyst in a 6-year-old boy and describes the treatment administered.

## RESUMEN

Los quistes dentígeros son una patología quística común que se desarrolla entre la primera y la tercera década de la vida, y se asocian principalmente con terceros molares mandibulares incluidos o erupcionados, seguidos de caninos superiores y terceros molares superiores. Este tipo de quistes son el resultado de la proliferación del epitelio del esmalte después de su formación, cuya patogenia no está clara. Se han informado pocos de estos quistes en pacientes pediátricos. El siguiente reporte de caso presenta la rara ocurrencia de un quiste dentígero en un niño de 6 años y describe el tratamiento administrado.

## INTRODUCTION

Different infectious lesions, benign and malignant tumors and cystic lesions can be found in the maxillofacial region. The latter are the most frequent in dentistry ^1^^-^^3^, the most prevalent being radicular cysts, which are part of the group of inflammatory cysts, followed by dentigerous cysts, which are developmental cysts ^(4, 5)^. The latter represent 20% of maxillary cysts and are generally associated with the crowns of unerupted teeth and can be observed in patients with a wide age range. In children they are infrequent, being found in 4 to 9% of the cases ^1^^,^^2^^,^^6^.

Dentigerous cysts are usually associated with mandibular third molars, canines and maxillary third molars ^5^^-^^7^. They are the result of the proliferation of the enamel epithelium after enamel formation. The pathogenesis of these cysts is not entirely clear, and they are thought to develop from abnormal accumulation of fluid between the enamel organ and the crown of the tooth ^4^^,^^8^^,^^9^.

Dentigerous cyst are clinically asymptomatic and are sometimes discovered on routine radiographic examination mainly by panoramic radiography. In the case of children, this type of study is often requested as an initial imaging examination to evaluate intraosseous formation of permanent teeth or an eruptive delay. However, some children may report discomfort in the buccal area, and in these cases, clinical and radiographic studies play a fundamental, and depending on the case, histopathological role ^4^^,^^10^^,^^11^.

The evolution of dentigerous cysts may be aggressive and extensive, leading to expansion or erosion of a bone table, displacement or reabsorption of neighboring dental germs, or the presentation of symptoms such as patient discomfort, among others ^(8, 10, 12)^. The treatment of choice is enucleation together with extraction of the tooth involved, but in pediatrics a more conservative approach involves marsupialization which is performed to save the affected tooth and allow correct eruption and alignment ^13^.

The need for an expanded initial imaging evaluation generally leads to a three-dimensional assessment with cone beam computed tomography (CBCT), an examination that has become a fundamental tool in dental treatment planning in recent years. It is a relatively low-cost test compared to a medical CT scan, but its main advantage is its low radiation levels. Taking into account that the need for its use is often focused on growing patients, it is important to apply the ALARA principle for its application.

This article describes the case of an aggressive dentigerous cyst in a pediatric patient as well as its respective treatment and histopathological diagnosis.

## CASE REPORT

A 6-year-old male patient attended a private dental clinic due to an increase in intraoral soft tissue volume in the retromolar area of the second lower left temporal molar. Intraoral clinical examination showed an increase in the volume of the soft tissues in the area referred, with an erythematous appearance, soft and painful on palpation, and without drainage on compression. On extraoral examination at the level of the left mandibular body, normal tissues were observed.

**Figure 1:**
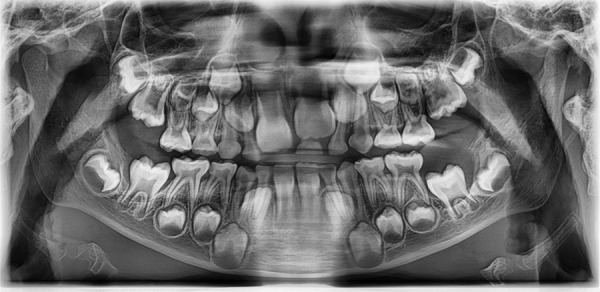
Panoramic radiography revealing an expansile radiolucent lesion related to the first molar in the left side.

Panoramic radiography showed a radiolucent image located at the crown of tooth 36 with defined limits and rounded in shape. There was an increase in the volume of the adjacent soft tissue in the cephalic direction and an increase in the surrounding bone density (Figure 1).

**Figure 2:**
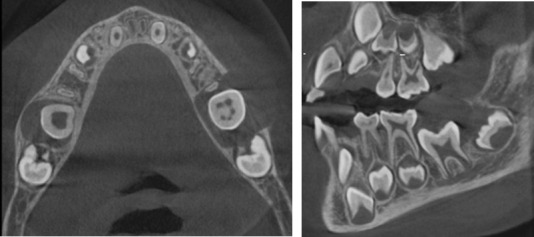
Axial and transaxial view in cone beam computed tomography (CBCT).

**Figure 3:**
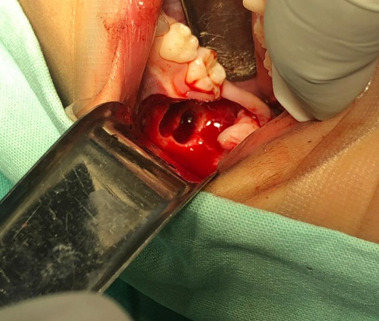
Intraoperative photography.

**Figure 4:**
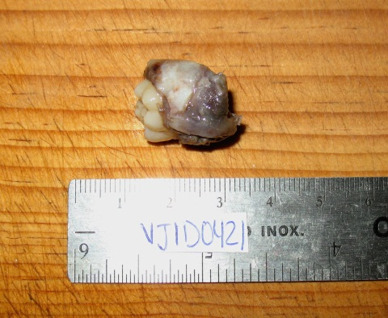
Extracted tooth with the cystic lesion.

Cone beam computed tomography (CBCT) showed an irregular hypodense area at the occlusal level of the germ of tooth 36, being unilocular and surrounded by a hyperdense line, with involvement of the germ of tooth 37, scalloping the adjacent dental structures and generating displacement of tooth 75 (Figure 2). Additionally, displacement and erosion of the vestibular bone table with thinning of the lingual bone table was observed, establishing a presumptive radiographic diagnosis of cystic lesion to rule out dentigerous cyst versus infected mandibular buccal cyst. In addition, the presence of a hypodense lesion with similar imaging characteristics was visualized in the contra lateral quadrant. With the above findings, the patient was referred to the oral and maxillofacial surgery service, in which the treatment plan was enucleation and curettage of the pathology, extracting the involved piece (Figures 3 and 4).

**Figure 5:**
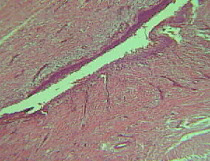
Histopahological section.

The histopathological study showed a cystic tumor lined all around by a completely uniform non-keratinized stratified flat epithelium. Likewise, a basal membrane formed by some squamous cells that did not present any involvement was observed, and no evidence of granulosa cells was observed on the surface of the wall. Additionally, the contour of the cystic structure of the tumor was not altered, and there were some areas slightly formed by mature fibroblasts. Finally, the histopathological diagnosis was established as a dentigerous cyst (Figure 5).

## DISCUSSION

Few studies have reported the presence of a dentigerous cyst in upper central incisors, or in mandibular first and second molars ^2^. These cysts mainly occur in the permanent dentition, although some cases have been reported in deciduous dentition ^9^. The evolution of dentigerous cysts is slightly faster in children compared to adults ^6^^,^^13^, and usually begins with a non-painful increase in volume in the affected area which may lead to future discomfort and even bacterial infection ^11^. Radiographically, dentigerous cysts are shown as a radiolucent lesion found in the crown of the tooth at the level of the enamel-cement junction, presenting well defined limits and sometimes with a corticalized border. CBCT usually shows expansion, thinning and erosion of the bone tables ^4^^,^^5^.

According to age there is a higher prevalence of onset between the first and third decade of life ^1^^,^^2^^,^^6^, although the frequency of onset is higher in the second decade. The prevalence in the first decade of life is low ^9^. Only a few studies have reported dentigerous cysts in the pediatric population. Suresh *et al*. ^12^ described the presence of a dentigerous cyst in a 1-year-old patient, while Kawamura *et al*. ^8^ reported one of these cysts in a 6-year-old male and Kalaskar *et al*. ^14^ described the presence of such a cyst in a 7-year-old boy. Microscopically the lumen of a dentigerous cyst is usually lined by non-keratinized stratified squamous epithelium that may vary in the presence of underlying infection resulting in hyperplasia of the lining epithelium and an increase in thickness. In the present study a cystic tumor was observed, which was lined all around by a completely uniform stratified flat non-keratinized epithelium.

The treatment followed in the present case was enucleation with curettage of the area and extraction of the tooth involved. Marsupialization was the treatment approach chosen, since it is a more conservative treatment ^8^^,^^13^, but complete removal is important to avoid future recurrences ^5^^,^^13^.

The precise imaging findings observed in the routine panoramic radiography complemented with CBCT allowed quick and effective treatment, without the need for with other radiographic studies which would have involved more exposure to radiation. This aspect is especially important to ensure radioprotection measures taking into account the age of the patient. Additionally, early detection reduces the risk of possible complications related to cyst size and involvement of anatomical structures. Sequelae produced by the presence of a dentigerous cyst have been reported in pediatric patients including amblyopia in a 4-year-old child ^15^ and diplopia in an 11-year-old child ^16^. Early and accurate presumptive diagnosis by an oral and maxillofacial radiologist is essential in cases such as that reported in the present study.

## CONCLUSIONS

• Early detection of a dentigerous cyst is important in the evaluation of a routine imaging examination as part of an early dental consultation. 

• Intraoral clinical aspects at early ages, such as tumefactions without apparent cause, or eruptive delays should be taken into account for requesting imaging studies such as routine panoramic radiography with low dose radiation. 

• The panoramic radiography can be complemented by a three-dimensional study such as CBCT with low doses of radiation, which provides sufficient information for adequate treatment decision making regarding the need or not for an immediate surgical approach. 

## Financing Sources:

Self-financed
